# Identification of an NFIA::CBFA2T3 fusion in cerebrospinal fluid confirms the diagnosis of pediatric CNS myeloid sarcoma with erythroid differentiation: a case report and literature review

**DOI:** 10.3389/fonc.2026.1676730

**Published:** 2026-05-12

**Authors:** Yang Zhao, Zhizhuo Huang, Lian Xue, Leping Zhang, Yueping Jia

**Affiliations:** Department of Pediatrics, Peking University People’s Hospital, Beijing, China

**Keywords:** cerebrospinal fluid, myeloid sarcoma with erythroid differentiation, NFIA::CBFA2T3, pediatric, *TP53*

## Abstract

Myeloid sarcoma with erythroid differentiation represents a mass-forming presentation of acute erythroid leukemia. This entity is exceptionally rare in the pediatric population, with only sporadic reports of *de novo* cases predominantly involving the central nervous system or orbit. Diagnosing myeloid sarcoma with erythroid differentiation poses significant clinical and pathological challenges, particularly in cases without bone marrow involvement. Here, we report the case of a 1-year-old boy with myeloid sarcoma with erythroid differentiation exhibiting diffuse parenchymal brain infiltration without mass formation. Due to the patient’s critical condition, a tissue biopsy was unfeasible. However, cerebrospinal fluid (CSF) flow cytometry revealed a significant population of immature erythroid cells, and RNA sequencing identified an NFIA::CBFA2T3 fusion—a genetic alteration previously reported in multiple myeloid sarcoma with erythroid differentiation cases. Notably, molecular testing confirmed that the patient was negative for both *TP53* mutation and chromosome 17 loss. Given the diagnostic complexity of this tumor, both flow cytometry and RNA sequencing played pivotal roles in establishing the definitive diagnosis.

## Introduction

Acute erythroid leukemia (AEL), previously known as pure erythroid leukemia, is a rare and distinct type of acute myeloid leukemia (AML) in the fifth edition of the World Health Organization classification system (1), whereas in the 2022 International Consensus Classification (ICC), AEL is predominately included within the category of “acute myeloid leukemia with mutated TP53” (2). In children, AEL can present with mass formation or involvement of body fluids, such as orbit (3, 4), ovaries (5), and especially the central nervous system (CNS) (6), with or without concurrent bone marrow involvement, so-called “myeloid sarcoma with erythroid differentiation”.

The diagnosis of AEL can be established by evaluating the characteristic pronormoblastic morphology, the variable expression of erythroid-specific antigens (hemoglobin and glycophorin A), and the presence of biallelic TP53 alterations, which are consistently associated with a complex or monosomal karyotype. However, in the pediatric population, AELs likely represent a distinctly different disease process from that of adults, harboring unique genetic features that appear to be enriched for rearrangements involving NUP98 (7) and NFIA.

In this study, we investigated the detailed clinicopathologic characteristics, diagnostic challenges, and therapeutic management of a unique case of CNS myeloid sarcoma with erythroid differentiation harboring an NFIA::CBFA2T3 fusion without bone marrow involvement. While this specific case was biologically characterized as part of a large genomic landscape cohort study by Fitzpatrick et al. (8), that report focused primarily on the molecular pathology of the disease entity. Here, we provide the first comprehensive description of the clinical presentation, cerebrospinal fluid (CSF) flow cytometry dynamics, and the specific multimodal therapeutic regimen that led to sustained remission. We also reviewed pediatric cases of myeloid sarcoma with erythroid differentiation retrieved from PubMed (http://www.ncbi.nlm.nih.gov/pubmed/) to summarize the major clinicopathologic features and prognosis (**Table 1**).

**Table 1:** Clinicopathologic features of pediatric erythroblastic sarcoma.

No.	Report	Age on set	Sex	Karyotype and FISH	Fusion	Treatment	Outcomes	mutation	Extramedullary Disease/site
1	King et al. (2021) (17)	2 mo	F	46,XX,der(1)t(1;8) (p31.3;q21.3)t(1;2) (p13;p23),der(2)t(1;2) t(1;8),der(8)t(1;8) (3)/47,sl, +mar (8)/46,sdl1,−X (8)/4 6,XX (1)	NFIA-RUNX1T1	AAML1031	/	KIT (NM_000222: c. 2446G-T: p,Asp816Tyr); ARID1A (NM_006015: c 570_573GGGC>TGT: p:Gly191fs*41)	Abdomen
2	Liu et al., 2020 (6)	2 y	F	54,XX,+X,t(1;16)(p31;q24),+ 6,+7,+8,+8,+10,+14,+19[1 2]/55,sl,+15 (8)	NFIA-CBFA2T3	AAML1031 w+BMT	Alive 28 months after diagnosis and treatment	EPOR (NM_000121.3:c.1316G>A:p. Trp439Ter) JAK2 (NM_004972.3:c.2651T>C:p. Leu884Pro) ARID1A (NM_006015.4:c.2231 C>G:p. Ser744Ter) ARID1A (NG_029965.1 (NM_006015.4):c.1920 + 9172_287 8 + 15del)	Brain
3	Micci et a 2011 (18)	15 mo	M	46,XY,der(1)t(1;1) (p31;q21),del(1) (p11p31),der(16)t(1;16) (p31;q24)	NFIA-CBFA2T3	NOPHO-AML 2004	Died 5 months after presentation and treatment	/	/
4	Castaneda et al 1991 (19)	6 y	M	51,XY, t(1;16)(p31;q24), +6, +10, +15, +19, +21	NFIA-CBFA2T3 ^a^	/	/	/	Abdominal wall
5	Koller et al., 1989 (20)	10 y	M	47,XY,t(1;16)(p31;q2?2),del(7) (q31),þ19,del(20)(p11)	NFIA-CBFA2T3 ^a^	/	/	/	Spinal mass
6	Alexandra L.et al 2025 (10)	10 days	M	46, XY	SREBF1:CIC	Low-dose cytarabine followed by high-dose cytarabine	Died 2 months after presentation and treatment	MYD88 R209C	Abdomen
7	Huan-You Wang 2011 (5)	3.5months	F	47,XX,del(6)(q23q25),+7 (21)	/	Pediatric Oncology Group protocol 9317	Alive 23 months after diagnosis and treatment	Deletion of C-MYB at 6q23	Ovaries
8	Razvan L 2016 (3)	3 months	M	(46, XY, del(6)(q25), add(19)(q13.3) (20)	/	Combination chemotherapy with alternating cycles of ifosfamide and etoposide, and cyclophosphamide, vincristine, and doxorubicin	Died 8 weeks after presentation and treatment	Deletion of C-MYB at 6q23	Orbit
9	Reena D 2016 (4)	1-year, 9-month-old	F	47,XX, t(11;20) (p15;p13), + add(17) (p13) (2)/ 47,sl, der(11)t(1;11) (q?21;q?24) (14)/47,sdl,add(8) (p23) (2))	/	BFM AML high-risk 1998 protocol	Alive	/	Orbit
10	Yevgeniy L 2020 (21)	3y	M	/	NFIA-CBFA2T3	/	Alive	LZTR1 (c.180C > A; p.C60 SMARCA4, CTCF, KMT2A, HIS T1H1E, and SLC26A3.	CNS mass
11	Obianuju M 2024 (22)	22 months	F	/	NFIA-CBFA2T3	Chemotherapy consisting of intravenous dexamethasone, cytarabine, cyclophosphamide	Alive	/	Parotid ,CNS
12	Tauziède-Espariat et al. 2024 (23)	3y	M	/	NFIA::RUNX1T1	Myechild 01 Trial	Died 3 months after presentation and treatment	/	CNS
13	Present case	1y	M	46,XY (20)	NFIA-CBFA2T3	AML-type induction chemotherapy+azacitidine+venetoclax	Alive 28 months after diagnosis and treatment	JAK2 (NM_004972 Exon 14:c.1832T>C:p.L611S), EPOR (NM_000121 Exon 8:c.1249G>T:p.E417X),ARID1A (NM_006015 Exon 34:c.1765C>T:p.Q589X)	CNS
14	Satoru Oya 2022 (9)	6 months	M	46,XY (20)	RCC1–LCK	Cytarabine, anthracyclines, and etoposide+allogeneic cord blood transplantation	Alive 20 months after cord blood transplantation	/	Humerus

a: Fusion presumed based on karyotypic findings, molecular studies not performed.

## Materials and methods

### Flow cytometric immunophenotyping

Samples of CSF were examined with flow cytometric immunophenotyping using two eight-color tubes containing antibodies from BD Biosciences (Tube1: TRBC1 FITC, CD15 cFluor548, TCRgd PE, CD117 pe-cy5, CD56 PE-Fire700, CD34 pe-cy7, CD3 cFluor R668, CD5 cFlour R720, CD38 APC-Fire750, CD19 BV421, CD7 BV480, CD45 BV510, CD16 BV570, HLA-DR BV605, CD33 BV650, CD10 BV711, CD4 BV750, CD123 BV785; Tube2: TDT FITC, CD64 PE, CD36 APC-CY7, CD45 BV510; Tube 3:CD42b APC, CD61+CD41a FITC, CD57 bv510, CD117 PE; and Tube 4: MPO FITC, CD235a PE, CD71 PE-Dazzle594, cCD79a APC, cCD3 AF647, CD105BV521, CD13 PE-CY7, EP+cyto eFlour 450). A total of 100,000 events were collected per case. The data were analyzed using Kaluza software (Beckman-Coulter, Brea, CA) and/or Diva software (BD Biosciences).

### Molecular genetic analysis

DNA was extracted from the CSF sample and following library preparation by hybrid capture, subjected to next-generation sequencing (NGS) on an Illumina NovaSeq 6000 Sequencing System (Illumina, Inc.) with post-sequencing analysis of tumor-associated mutations. The performance characteristics of the NGS panel were as follows: for single-nucleotide variants (SNVs), accuracy >99%, intra- and inter-assay reproducibility 100%, and sensitivity of 5%–10% variant allele frequency at a minimum coverage depth of 250×; for insertion/deletion (indel) events, accuracy >99%, intra- and inter-assay reproducibility 100%, and sensitivity of 5–10% variant allele frequency at a minimum coverage depth of 250×.

## Case presentation

### Clinical history and initial evaluation

This patient was a previously healthy 1-year-old boy **who** presented to the pediatric department with intermittent fever, epileptic seizures, and subsequent left-side hemiplegia. Physical examination showed the presence of **a** setting-sun phenomenon and significantly increased fontanel tension. Initial complete blood count (CBC) demonstrated mild normocytic anemia (hemoglobin 10.2 g/dL; reference range: 12.0–14.0 g/dL) and normal platelet count (256×10^9^/L; reference range: 100–300×10^9^/L) without evidence of circulating blasts (<1% on peripheral smear). Serum studies showed preserved end-organ function including lactate dehydrogenase (LDH), hepatic transaminases, bilirubin, and serum creatinine, all within normal limits. Coagulation parameters revealed hypofibrinogenemia (158 mg/dL; reference 200–400 mg/dL) and a markedly elevated D-dimer level (2,927 ng/mL; reference 0–243 ng/mL), whereas prothrombin time (12.0 s; reference 9.4-12.5 s), activated partial thromboplastin time (24.1 s; reference 25.1-36.5 s), and international normalized ratio (1.07; reference 0.9-1.2) were within normal limits. These coagulation parameters did not fulfill the diagnosis for disseminated intravascular coagulation (DIC) according to the International Society on Thrombosis and Haemostasis (ISTH) scoring criteria (DIC score < 5). Bone marrow aspirate revealed normocellular hematopoiesis with trilineage maturation and no morphologic or immunophenotypic evidence of blast proliferation.

### Diagnostic imaging and CSF analysis

**Figure 1:**
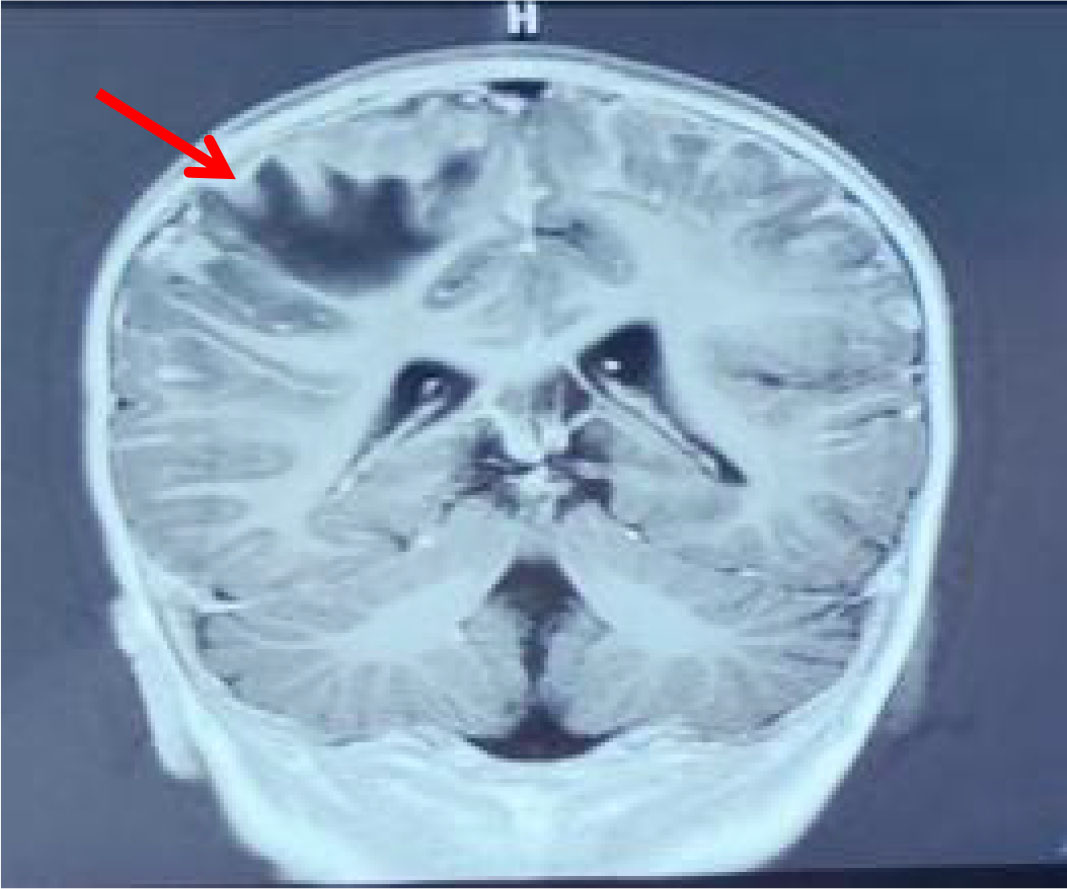
Cranial MRI revealed bilateral parietal parenchymal swelling and hypointense white matter lesions in the right parietal and frontal lobes.

Electroencephalography (EEG) demonstrated diffuse polymorphic delta slowing (2–3 Hz) with loss of posterior dominant rhythm, consistent with a generalized encephalopathic pattern without epileptiform discharges. The cranial magnetic resonance imaging (MRI) revealed bilateral parietal parenchymal swelling and hypointense white matter lesions in the right parietal and frontal lobes, but without mass formation (**Figure 1**). Tissue biopsy could not be performed due to the absence of a discrete cranial mass and the patient’s unstable clinical status. After administration of intravenous mannitol to reduce elevated intracranial pressure, a diagnostic lumbar puncture was successfully performed.

CSF was clear and colorless. Analysis demonstrated a strongly positive Pandy’s test and pleocytosis (113×10^6^/L leukocytes; reference range: 0–15×10^6^/L) with a differential of 83% mononuclear and 17% polymorphonuclear cells. Biochemistry revealed markedly elevated protein (14,668 mg/L; reference range: 200–400 mg/L) with normal levels of glucose, chloride, lactate, lactate dehydrogenase (LDH), and adenosine deaminase (ADA).

### Cytological and flow cytometric assessment

**Figure 2:**
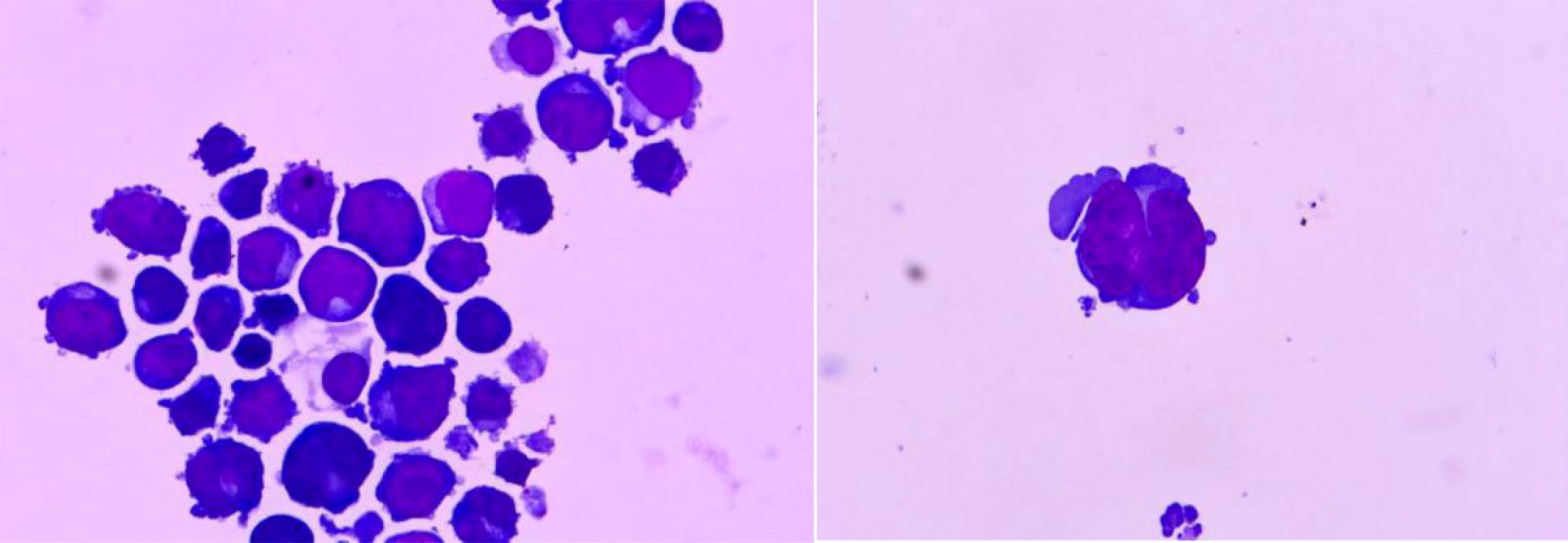
Medium-large size erythroid lineage precursors with irregular nuclei (coarse chromatin/nucleoli), basophilic blebbed cytoplasm, and binucleated cells.

**Figure 3:**
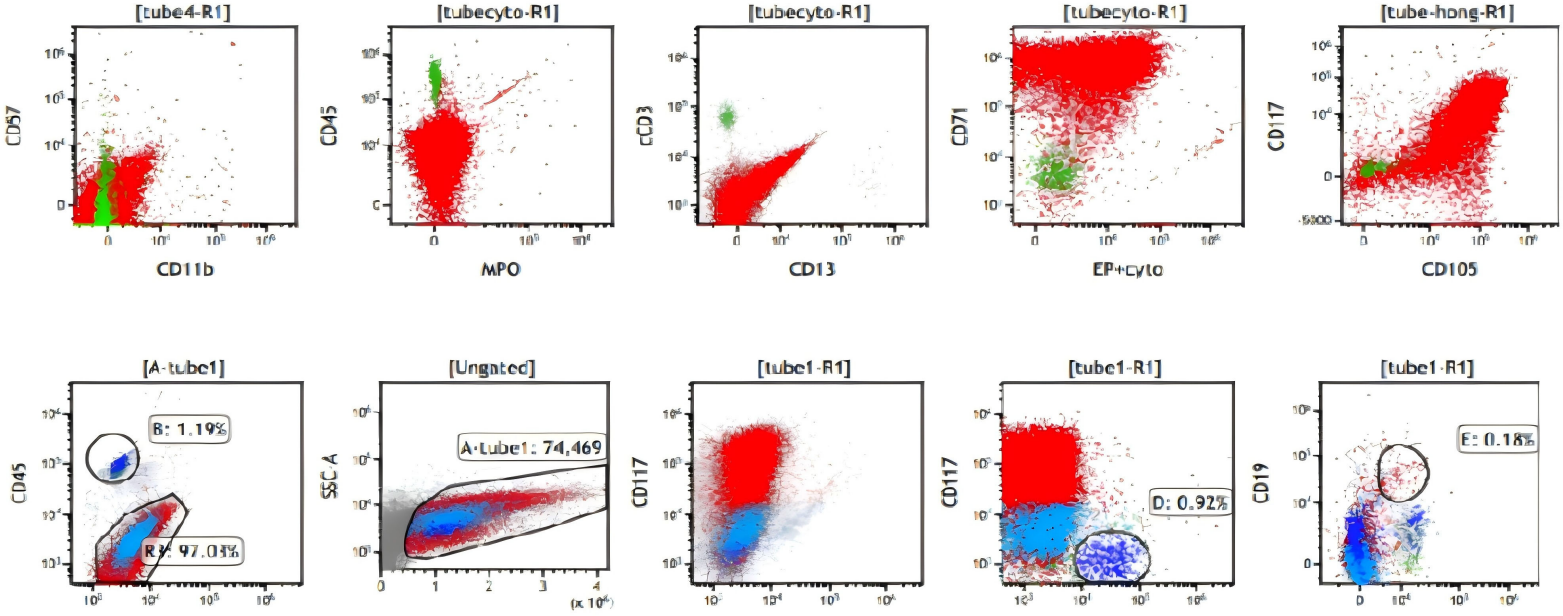
Flow cytometry demonstrated CD71+/CD43+/CD117+/CD45-.

Cytological examination identified a total nucleated cell count >100, of which 91% were blasts (medium-large size with irregular nuclei, coarse chromatin, and basophilic blebbed cytoplasm) (**Figure 2**). Given the diagnostic ambiguity, CSF samples were analyzed via flow cytometric immunophenotyping (as detailed in the Materials and Methods section). CSF flow cytometry revealed a predominant CD45-negative cell population (97.03%) strongly expressing erythroid lineage-specific antigens (CD71, CD36, CD105, CD235a) and the stem cell marker CD117, while lacking myeloid (MPO, CD13, CD33) and lymphoid markers. This immunophenotype (CD45-/CD117+/CD71+/CD235a+) was highly suggestive of erythroblasts and concerning for CNS myeloid sarcoma with erythroid differentiation (**Figure 3**).

### Molecular genetic analysis

CSF analysis revealed negative conventional microbiological stains, negative enterovirus PCR, and no pathogen detection by metagenomic NGS. Due to the absence of tumor tissue for biopsy and the rarity of the suspected diagnosis, a comprehensive multi-omics approach was adopted to identify the oncogenic driver.

**Figure 4:**
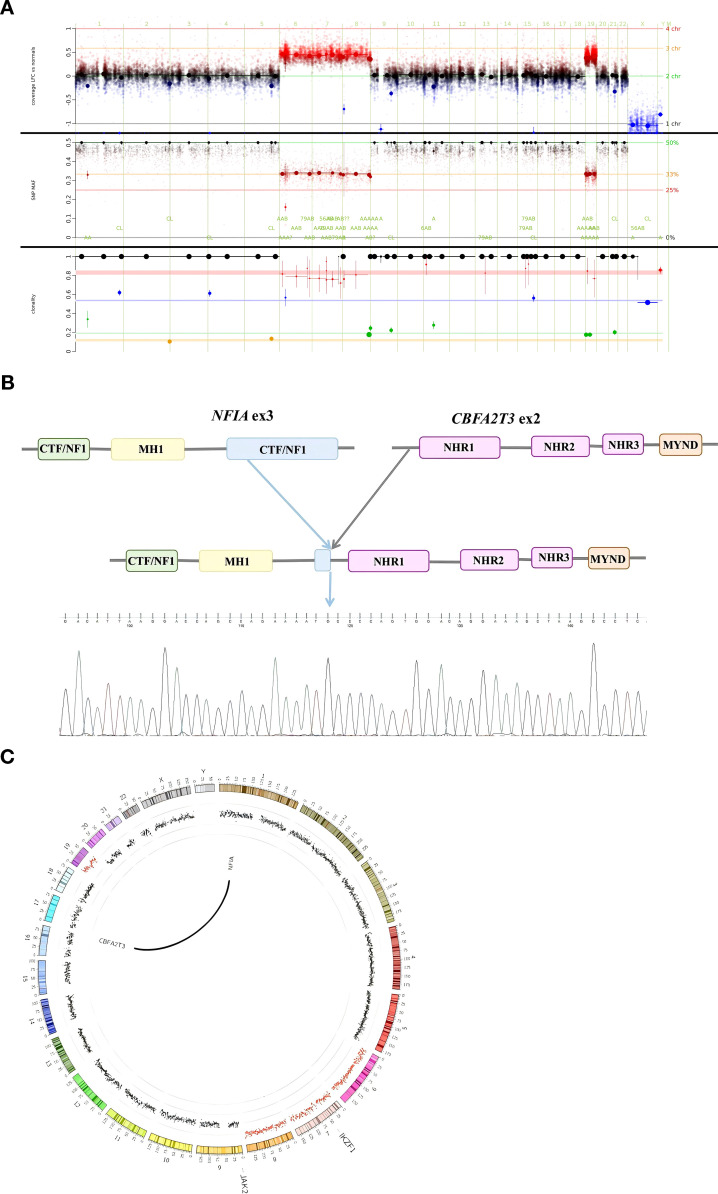
Molecular landscape of the tumor. **(A)** WGS copy number profile showing gains of chromosomes 6, 7, 8, and 19. **(B)** Sanger sequencing chromatogram of the RT-PCR product confirming the specific nucleotide sequence at the junction point of the NFIA exon 3 and CBFA2T3 exon 2 fusion. **(C)** RNA-Seq analysis output (Sashimi plot) visualizing the NFIA::CBFA2T3 fusion reads.

Whole-genome sequencing (WGS) demonstrated recurrent numerical chromosomal abnormalities involving gains of chromosomes 6, 7, 8, and 19 (**Figure 4A**). Whole-exome sequencing (WES) identified clinically significant genomic alterations including somatic variants in JAK2 (NM_004972 Exon 14:c.1832T>C:p.L611S), EPOR (NM_000121 Exon 8:c.1249G>T:p.E417X), and ARID1A (NM_006015 Exon 34:c.1765C>T:p.Q589X) and a germline variant in JAK2 p.G127D. Notably, comprehensive genetic testing confirmed the complete absence of a *TP53* mutation and loss of chromosome 17.

Crucially, RNA-sequencing demonstrated an NFIA::CBFA2T3 fusion, fusing exon 3 of NFIA in-frame to exon 2 of CBFA2T3 (**Figure 4C**). This result was verified by reverse-transcriptase PCR (RT-PCR) targeting the fusion junction, followed by Sanger sequencing of the resulting amplicon (**Figure 4B**). Published literature indicates that a similar NFIA::CBFA2T3 fusion is characteristically observed in pure erythroid leukemia/erythroid sarcoma (PEL/ES), and this genetic rearrangement has been established as a characteristic molecular marker for this disease entity. The chimeric fusion protein was predicted to cause partial truncation of the C-terminal CCAAT box-binding transcription factor (CTF)/nuclear factor I (NFI) domain in NFIA (NP_001128145), while maintaining the conserved TAF and nervy homology (TAFH and NHR2) domains within CBFA2T3 (NP_005178) (6).

### Therapeutic intervention and outcomes

Following the definitive diagnosis of CNS myeloid sarcoma with erythroid differentiation, AML-type induction chemotherapy (7 + 3 regimen with idarubicin/cytarabine) was initiated. Given the life-threatening cerebral herniation at disease onset, cytarabine was initiated at a minimal dose and gradually up-titrated based on the patient’s tolerance (50mg/m^2^ d1; 75mg/m^2^ d2-3, 1,000mg/m^2^ d4-7), in combination with the hypomethylating agent azacitidine (75mg/m^2^, d3-7) and concurrent oral venetoclax (125mg/m^2^, d3-10) to promote tumor cell apoptosis. The pediatric patient demonstrated good chemotherapy tolerance with **a** gradual intracranial pressure reduction. Follow-up head CT on day 10 of treatment revealed significant improvement in the previously noted right parieto-frontal cortical hyperdensities with white matter edema and cerebral herniation. Post-therapeutic lumbar puncture was performed. CSF analysis demonstrated normal routine parameters and biochemical profile, flow cytometry was 61.81% positive for malignancy, and analysis for the NFIA::CBFA2T3 fusion gene was 2.16% positive.

The pediatric patient successfully completed 11 cycles of AML-type consolidation chemotherapy in combination with a hypomethylating agent (azacitidine 75 mg/m^2^/day subcutaneously on days 1-7) and the BCL-2 inhibitor venetoclax (200mg/m^2^/day subcutaneously on days 1-14) and received a total of 14 prophylactic intrathecal therapies (methotrexate 7.5mg/cytarabine 20mg/m^2^/dexamethasone 5mg). The specific chemotherapeutic regimen and dosages are detailed in **Table 2**. Serial CSF analysis and monthly bone marrow evaluations demonstrated sustained complete remission (CR). He currently remains in complete remission (27 months after initial presentation).

**Table 2:** The specific chemotherapeutic regimen and dosages of this patient.

Systemic therapy by cycle	Drug, dose, route, and day schedule	Intrathecal treatments (methotrexate 7.5mg+cytarabine 20mg/m^2^+dexamethasone 5mg)	CSF response assessments	BM response assessments
Induction chemotherapy	Cytarabine iv. (50mg/m^2^ d1;75mg/m^2^ d2-3,1,000mg/m^2^ d4-7)+idarubicin iv.(8mg/m^2^ d2-3)+azacitidine iv.(75mg/m^2^,d3-7) +venetoclax po.(100mg/m^2^,d3-10)	For four times	First CSF routine parameters and biochemical profile: (−), flow cytometry:61.81% positive ; *NFIA::CBFA2T3* = 2.16%. Subsequent CSF flow cytometry:(−), *NFIA::CBFA2T3* = 0%	Complete remission(CR)
Consolidation block 1	Azacitidine iv.(75mg/m^2^,d1-7) +Cytarabine iv. (1,000mg/m^2^ d1-3)+venetoclax po.(100mg/m^2^, d1-14)	For one time	CSF routine parameters and biochemical profile: (−), CSF flow cytometry:(−), *NFIA::CBFA2T3* = 0%	CR
Consolidation block 2	Azacitidine iv.(75mg/m^2^,d1-7) +idarubicin iv.(8mg/m^2^ d2-3)+venetoclax po.(100mg/m^2^, d1-14)	For one time	CSF routine parameters and biochemical profile: (−), CSF flow cytometry:(-), *NFIA::CBFA2T3* = 0%	CR
Consolidation block 3	Azacitidine iv.(75mg/m^2^,d1-7) +cladribine iv. (5mg/m^2^ d1-5)+venetoclax po.(100mg/m^2^, d1-14)	For one time	CSF routine parameters and biochemical profile: (−), CSF flow cytometry:(−), *NFIA::CBFA2T3* = 0%	CR
Consolidation block 4	Azacitidine iv.(75mg/m^2^,d1-7) +cytarabine iv. (1,000mg/m^2^ d1-3)+venetoclax po.(100mg/m^2^, d1-14)	For one time	CSF routine parameters and biochemical profile: (−), CSF flow cytometry:(−), *NFIA::CBFA2T3* = 0%	CR
Consolidation block 5	Azacitidine iv.(75mg/m^2^,d1-7) +idarubicin iv.(8mg/m^2^ d2-3)+venetoclax po.(100mg/m^2^, d1-14)	For one time	CSF routine parameters and biochemical profile: (−), CSF flow cytometry:(−), *NFIA::CBFA2T3* = 0%	CR
Maintenance chemotherapy	Azacitidine iv.(75mg/m^2^,d1-7) +venetoclax po.(100mg/m^2^, d1-28), for a total of six cycles	For five times	CSF routine parameters and biochemical profile: (−), CSF flow cytometry:(−), *NFIA::CBFA2T3* = 0%	CR

## Discussion

The diagnosis of AEL relies on characteristic pronormoblastic morphology, variable erythroid antigen expression (hemoglobin and glycophorin A), and the invariable presence of biallelic TP53 alterations associated with complex/monosomal karyotypes. The classification of its extramedullary, mass-forming counterpart-erythroblastic sarcoma (ES) highlights a conceptual divergence between the two major contemporary classification systems. The proposed WHO-HAEM5 classifies AEL under “Myeloid/Lymphoid Neoplasms with Erythroid Differentiation”. Accordingly, ES is regarded as the extramedullary tumoral manifestation of the same disease, implying a direct clinicopathologic continuum. In contrast, the ICC 2022 incorporates cases historically classified as AEL into the genetically defined category of “AML with mutated TP53,” reflecting the near-universality of complex karyotypes and TP53 alterations in these neoplasms. Under the ICC, an isolated extramedullary proliferation of pronormoblasts is diagnostically framed as a myeloid sarcoma with erythroid differentiation, further subclassified based on the presence or absence of systemic (marrow) involvement by TP53-mutated AML.

However, the index case presented in this paper lacked both a *TP53* mutation and loss of chromosome 17. The 2022 ICC diagnostic criteria heavily rely on *TP53* mutations for the classification of AEL, which aligns accurately with the molecular landscape of adult cases but presents a significant diagnostic challenge for pediatric patients. As demonstrated by our case and the existing literature, pediatric myeloid sarcoma with erythroid differentiation is predominantly driven by distinct structural variants, such as the NFIA::CBFA2T3 fusion, rather than *TP53* mutations or chromosome 17 abnormalities. This discrepancy highlights the limitation of applying the strict ICC classification to pediatric populations and underscores the necessity of recognizing pediatric erythroblastic sarcoma as a distinct molecular entity. Therefore, our case should be described as CNS myeloid sarcoma with erythroid differentiation, consistent with WHO-HAEM5’s focus on extramedullary myeloid neoplasms, supported by morphology and immunophenotype indicating erythroid sarcoma.

Myeloid sarcoma with erythroid differentiation was first delineated by Wang et al (5). Systematic review identifies 20 pediatric cases including our index case until July 2025, and seven cases including our case represented a distinct molecular entity characterized by recurrent NFIA::CBFA2T3 fusions. Other fusions include NFIA::RUNX1T1, RCC1::LCK (9), and SREBF1:CIC (10). Notably, nearly all recurrent rearrangements (93% in reported cases) are detected in patients ≤3 years old, suggesting infantile myeloid sarcoma with erythroid differentiation may represent a distinct clinicopathologic entity with unique molecular pathogenesis.

Pediatric myeloid sarcoma with erythroid differentiation demonstrates unique anatomic predilection characterized by frequent CNS involvement, head/neck region infiltration, and midline skeleton and soft tissues (**Table 1**). Distinct from previously reported cases, our patient presented with primary CNS involvement manifesting as diffuse parenchymal infiltration rather than a discrete mass lesion. This atypical radiologic phenotype posed significant diagnostic challenges and increased risk associated with invasive tissue biopsy. Like our case, CSF flow cytometry may serve as the critical diagnostic modality for early disease detection. Proerythroblasts demonstrate a characteristic immunophenotype characterized by complete absence of myeloid differentiation markers (CD34/HLA-DR/CD13/CD33/MPO), universal negativity for pan-leukocyte CD45, and lymphoid lineage antigens (CD19/CD20/CD3), while exhibiting strong co-expression of erythroid lineage-specific antigens including CD71, CD36, and CD235a.

Emerging evidence demonstrates that pediatric myeloid sarcoma with erythroid differentiation is predominantly driven by recurrent gene rearrangements, most frequently involving NFIA fusions with CBFA2T3 (historically termed ETO2 or RUNX1T3). In our case, whereas WGS and WES identified multiple chromosomal gains (+6, +7, +8, +19) and somatic mutations in JAK2 and EPOR, we posit that the NFIA::CBFA2T3 fusion is the primary oncogenic driver defining this entity. The chromosomal patterns observed (trisomy 8, etc.) are common non-specific findings in myeloid malignancies. However, the JAK2 p.L611S mutation is noteworthy. While JAK2 mutations are classic in myeloproliferative neoplasms, their co-occurrence with NFIA fusions suggests a potential cooperative mechanism where signaling pathway activation (JAK2/EPOR) complements the differentiation block induced by the fusion transcription factor. This highlights the utility of comprehensive sequencing (WES/RNA-seq) not only for diagnosis but for identifying potential therapeutic targets (e.g., JAK inhibitors) in refractory cases.

NFIA encodes a critical determinant directing the nuclear factor I (NFI) transcription factor family that mediates transcriptional regulation at the erythroid–myeloid bifurcation point, with upregulation of NFIA leading to erythroid differentiation and downregulation favoring myeloid development. NFIA also **acts** as a regulator that directly targets downstream effectors of erythroid differentiation (e.g., β-globin) and suppresses the transcriptional activity of granulocytic differentiation factors such as the granulocyte colony-stimulating factor receptor (11). CBFA2T3 (also known as ETO2) encodes a member of the ETO family of myeloid translocation gene proteins that functions as a transcriptional corepressor **by** interacting with DNA-binding transcription factors and recruiting multiple corepressors to mediate transcriptional repression (12). Functional studies demonstrate that the NFIA::CBFA2T3 fusion variant significantly enhances proliferation while impairing erythroid differentiation in both murine erythroleukemia (MEL) cells and primary fetal liver-derived erythroblasts (13).

The primary treatment for AEL follows myeloid-type regimens, including standard induction chemotherapy protocols such as DA (daunorubicin + cytarabine), HAD (homoharringtonine + cytarabine + daunorubicin) and MAE (mitoxantrone + cytarabine + etoposide). With advancing understanding of the disease, current research focuses on evaluating the efficacy of hypomethylating agents (HMAs) in TP53-mutated leukemia. Reichard et al. demonstrated that the HMA+venetoclax was the most effective regimen in achieving inducing CR/CRi (14). Venetoclax promotes apoptosis in chemotherapy-resistant tumor cells, whereas azacitidine enhances chemotherapeutic efficacy through epigenetic modulation. Due to the patient’s young age and limited tolerance, low-dose venetoclax was administered (serum concentration 800-980 ng/mL). Given the substantially lower CSF penetration of venetoclax and its uncertain efficacy in primary CNS erythroid neoplasms, the therapeutic strategy included repeated intrathecal therapy (15). The patient in our case ultimately achieved complete remission, demonstrating that this multimodal combination strategy was both effective and clinically instructive. Our patient presented with critical illness at disease onset, manifesting with cerebral herniation and status epilepticus upon admission. Following initiation of intracranial pressure reduction and midazolam infusion for seizure control, chemotherapy was promptly initiated after obtaining parental consent. In addition to appropriate chemotherapeutic regimen selection, rapid treatment initiation proved crucial for patient survival.

Previous studies have reported that AEL is associated with a poor prognosis, with median survival typically being less than 6 months (16). Outcome was favorably influenced by autologous hematopoietic stem cell transplantation (ASCT) and not by TP53 variant allele frequency (14). This pediatric case achieved sustained survival of 28 months with chemotherapy alone. Given excellent local control achieved with multi-agent intrathecal therapy, the patient’s young age, the inherent toxicities of allogeneic hematopoietic cell transplantation (allo-HCT), and parental preference, allo-HCT was not performed. Nevertheless, allo-HCT remains strongly indicated for patients with myeloid sarcoma carrying TP53 mutations, those failing to achieve deep molecular remission after intensive chemotherapy, or individuals otherwise deemed at very high risk of relapse. For such rare tumors, personalized treatment combining targeted agents (e.g., potential JAK inhibitors as suggested by the JAK2 mutation in this case) with chemotherapy based on underlying molecular mechanisms may represent a future therapeutic direction.

In conclusion, we report a rare pediatric case of CNS myeloid sarcoma with erythroid differentiation harboring an NFIA::CBFA2T3 fusion gene identified by RNA sequencing. Based on existing literature, we propose this fusion gene as a defining molecular feature of a distinct subtype of myeloid sarcoma with erythroid differentiation. This discovery not only enhances our understanding of ES pathogenesis but also provides a novel molecular marker and potential therapeutic target for precise diagnosis and targeted therapy of such rare tumors. We recommend routine molecular profiling, including RNA sequencing, for all primary CNS erythroid tumors to detect this fusion gene, complemented by flow cytometric analysis of proerythroblasts to facilitate accurate diagnosis, guide personalized treatment, and ultimately improve patient outcomes.

## Data Availability

The original contributions presented in the study are included in the article/supplementary material. Further inquiries can be directed to the corresponding author.
